# Association of eHealth Use, Literacy, Informational Social Support, and Health-Promoting Behaviors: Mediation of Health Self-Efficacy

**DOI:** 10.3390/ijerph17217890

**Published:** 2020-10-28

**Authors:** MoonKi Choi

**Affiliations:** College of Nursing, Kangwon National University, Chuncheon 24341, Korea; mkchoi@kangwon.ac.kr; Tel.: +82-33-250-8882

**Keywords:** eHealth, health literacy, health promotion, self-efficacy, social support

## Abstract

This descriptive, cross-sectional study identified the association of eHealth use, literacy, informational support, and health-promoting behaviors among older adults, as mediated by health self-efficacy. Convenience sampling was conducted at senior welfare centers in Chuncheon, in the Republic of Korea. Data analysis was performed using Pearson’s correlation and via path analyses. The findings showed that eHealth use had an indirect effect on health-promoting behaviors, as mediated by self-efficacy. Informational support was indirectly mediated by self-efficacy and had direct effects upon health-promoting behaviors. eHealth can facilitate self-efficacy and health management, despite not having direct effects upon health-promoting behaviors themselves. Thus, older adults need to be prepared for the increased use of eHealth. In addition, healthcare professionals should support older people in their use of eHealth and encourage informational support through comprehensive interventions so as to facilitate self-efficacy and health behaviors.

## 1. Introduction

In 2019, 703 million people around the world were older adults above the age of 65—a figure which is anticipated to double to approximately 1.5 billion by 2050 [1]. With the onset of aging, older adults are at increased risk of developing complex chronic diseases, and often develop at least two such conditions throughout their advanced years. Given the increased prevalence of chronic diseases among the older population, the social cost and burden of such diseases are high [2]. Thus, there has been a growing interest in health promotion so as to prevent disability and dependence among older adults [3].

Pender’s Health Promotion Model, which is based on social cognitive theory, is widely used in facilitating healthy behaviors and an overall improvement in health. According to the model, individual factors and experiences influence behavior-specific cognitions, such as self-efficacy and behavioral outcomes [4]. Self-efficacy is related to changes to one’s behavior so as to achieve desirable outcomes and further refers to the individual’s beliefs concerning how well one can execute the behaviors required to attain specific outcomes [5]. People with a high degree of self-efficacy believe that they are highly capable and, consequently, accept challenging and interesting tasks; people with low degrees of self-efficacy are, conversely, uncertain about their capacity to succeed and to achieve their goals. The association between self-efficacy and health-promoting behaviors has been supported by previous studies based on Pender’s Health Promotion Model, which uncovers factors affecting behavioral outcomes [6,7]. A meta-analysis showed that changes in behavior-specific cognition—such as self-efficacy—led to increased health-related intentions and behaviors [6] as self-efficacy affects an individual’s decisions and one’s subsequent actions as they relate to health enhancement [8]. Thus, it is important to identify the factors influencing both health self-efficacy and health promotion.

Internet usage is increasing as technology develops further. As such, although young people are more likely to use the Internet than their older counterparts, Internet usage is, nonetheless, growing in prominence among older generations. According to a nationally representative survey of the United States, 62.6% of individuals aged 65 to 74 years old made use of the Internet in 2011, about twice the percentage eight years ago (33.7%); Internet use also increased from 16.5% to 33.6% among the respondents above the age of 75 [9]. Various health-related interventions that are based on Internet connectivity or usage have been developed; this trend has been amplified by the growing ubiquity of smartphones, which can be linked with various biometric devices [10]. Going forward, eHealth is expected to play an important role as a healthcare tool for people with chronic illnesses; however, an increase in Internet usage does not readily translate to the use of eHealth aides among older adults.

eHealth is defined as a web-based technology that enables health-related work and includes a wide range of information technologies—such as health information exchange, healthcare software, and electronic appointment scheduling [11]. As smartphone use increases, eHealth is also combined with mHealth (mobile health)—which is healthcare support provided via mobile phones [11]. Recently, people have begun to expect a healthy, independent life and not merely a disease-free existence. Furthermore, older adults expect to “age in place” by receiving healthcare in their own homes, regardless of time and place. Thus, in parallel with these expectations, eHealth is anticipated to play a bigger role in healthcare interventions in the future [12]. Previous studies have reported that older adults expected convenient and easy healthcare as facilitated via eHealth—that is, increased access to medical information, the provision of interpersonal support among patients, the checking of prescriptions, and communication with medical staff [12,13]. eHealth applications in daily life can continuously provide older populations with access to the latest health information and motivate health-promoting behaviors; this, in turn, can lead to lifestyle changes [14].

According to reports, the use of health information derived from the Internet is increasing. More than 70% of US adults first make use of the Internet to search for health information, when needed [15]. However, in the case of eHealth, there is a notable risk of exposure to unreliable health information, a difficulty in identifying the correct information available online, or the misuse of this health information—all of which may have adverse effects [16]. Health literacy is defined as “the development of the cognitive and social skills which determine the motivation and ability of individuals to gain access to, understand and use information in ways that promote and maintain good health” [17]. eHealth literacy is required for the ability to search, understand, and evaluate reliable health information available via the Internet. Older adults may not be able to find the necessary health information on the Internet or understand the information available due to decreased sensory and cognitive functions, as well as due to the result of inefficient operation of the device [13]. It may also be difficult for older adults to make use of eHealth resources due to a difficulty in reading small text, understanding difficult icon shapes, and interpreting challenging device manipulations. Even though the interest in eHealth has been growing in older adults, those who with low levels of eHealth literacy may, as a result, avoid using eHealth resources due to a fear of accessing and selecting inaccurate or unreliable information, or in fear of misunderstanding the health-related material which they are able to access. Low eHealth literacy can, thus, compromise the effectiveness of eHealth services and health promotion.

Not all older adults prefer and use eHealth resources; digital divide-related health can occur according to age, ethnicity, Internet accessibility, and a preference of traditional methods of healthcare [18]. Considering web-based healthcare is being strengthened, the utilization of eHealth is expected to affect health-promoting behaviors both directly and indirectly among older adults. As the ability to find and understand health information is associated with health management processes [19], eHealth literacy can also be associated with health-promoting behaviors.

In addition, an individual’s social environment also plays an important role in health management; significant others can offer information, encourage healthy behavior, and provide a good example of active aging [20]. In social contexts, changes in behavior follows a cognitive process; thus, social support, and especially informational support, can assist older adults in facilitating self-efficacy and improved health behaviors. Informational social support refers to actions such as advice, suggestions, and instructions for solving an immediate problem, providing access to a variety of information, and helping to apply changes [21]. Older adults who receive actual or indirect help from people close to them—such as family and friends—may have a more trusting perception of aspects of healthcare and may better adhere to health-promoting behaviors. As the availability for help—whether it be face-to-face or online assistance—can supplement eHealth literacy and lead to positive health outcomes [22], subsequently, informational social support will be associated with self-efficacy and health-promoting behaviors among older adults.

This study identified the level of eHealth use, eHealth literacy, and informational social support among older Koreans and examined the relationship between these variables and health-promoting behaviors as mediated by health self-efficacy. The results of this study may provide evidence for the development of Internet-based interventions and, thus, help determine the role of health professionals in improving the health of the older population.

## 2. Materials and Methods

### 2.1. Research Design

This research was conducted via a descriptive study, using a cross-sectional survey design that aimed to identify the relationships between the level of eHealth use and literacy, and health-promoting behaviors among older community-dwelling Korean adults.

### 2.2. Sample

Using convenience sampling, 198 participants were recruited from two senior welfare centers in Chuncheon, Korea. Participants above the age of 65 years, who were able to self-report in Korean, and who were not diagnosed with dementia were included. Data were collected using structured questionnaires between September and December 2019. Participants filled out questionnaires by themselves; if necessary, the trained research assistants read the questions to the participants and marked their responses on the questionnaires. Data from 186 participants were used for statistical analysis; 12 participants were excluded due to missing values.

### 2.3. Measures

Information on the instruments for each variable is shown in Table 1. Personal characteristics—including age, sex, marital status, occupational status, education level, and subjective economic status—were investigated.

The respondents’ medical history, perceived health status, and cognitive functioning were also investigated. A respondent’s medical history—which includes the presence of conditions such diabetes, hypertension, dyslipidemia, and arthritis—was identified by asking what the participant had previously been diagnosed with. The respondent’s perceived health status was evaluated using a self-rated, single question with a 5-point Likert scale, whereby a higher score indicates better health. Cognitive functioning was measured by trained research assistants using the Korean version of the Mini-Mental State Examination (MMSE), as developed by Folstein, Folstein, and McHugh [23]. The MMSE is used to estimate the severity of cognitive impairment, including registration (repeating named prompts), attention and calculation, recall, execution and orientation, and language. A respondent’s total score range from zero to 30, with higher scores indicating better cognitive functioning.

The level of eHealth use was measured using categories of Internet health information-seeking behaviors [24]. The tool contains 13 items that inquire about production activities—such as writing on online bulletin boards, the use of health information communities, and searching for health information. The tool’s internal consistency, as a measure of Cronbach’s alpha, has been reported to be 0.90. Each item was scored on a 5-point Likert scale, with higher scores indicating an increased use of eHealth resources.

eHealth literacy levels were measured with the eHealth Literacy Scale (eHEALS), as developed by Norman and Skinner [25] and revised by Chang et al. [26]. The tool contains eight questions which evaluate an individual’s ability to search the Internet for health information, utilize it, and make decisions using the information found. The test–retest reliability and internal consistency of eHEALS, using Cronbach’s alpha, have been reported to be 0.80 and 0.89, respectively. In addition, confirmatory factor analysis showed that the eHEALS has good validity, with an acceptable model fit (Goodness of Fit Index = 0.94; Turker-Lewis Index = 0.93). Each item was scored on a 5-point Likert scale, with higher scores indicating better levels of eHealth literacy.

Informational social support was evaluated using the Social Support Scale, as developed by Park [27]. The tool includes four sub-concepts: emotional support, informational support that provides information that can be used to address personal problems, material support (including monetary or material help), and evaluative support (such as acknowledgment or respect). Park reported that the Social Support Scale showed a high internal consistency (Cronbach’s alpha = 0.94) and acceptable criterion validity. Of the 25 items, this study used four of items regarding informational support. Each item was scored on a 5-point Likert scale. Higher scores indicated a higher perception of social support.

Health self-efficacy was measured with Self-Rated Abilities for Health Practices Scale developed by Becker et al. [28] and revised by Lee et al. [29]. The tool contains 24 items assessing the efficacy of exercise, disease management, emotional health, nutrition, stress management, and health behavior. The tool has been reported to have a high internal consistency (Cronbach’s alpha = 0.91) and good criterion validity. Each item was evaluated on a 5-point Likert scale, where higher scores indicate higher degrees of efficacy of health care, culminating in a total possible score of 120 points.

Health-promoting behaviors were evaluated using the Health-Promoting Lifestyle Profile II, as developed by Walker, Sechrist, and Pender [30] and translated into Korean by Seo and Hah [31]. The tool comprises 52 items across 6 subscales: health responsibility, physical activity, nutrition, spiritual growth, interpersonal relations, and stress management. Each item was scored on a 4-point Likert scale; an overall score was obtained by calculating the mean of the individual’s responses to all items. Higher scores indicated better health-promoting behaviors.

### 2.4. Ethical Considerations

The Institutional Review Board (no. 2019-07-007-001; date. 22 October 2019) at a university in Korea approved this study. Prior to the collection of data, the study’s purpose and procedures were explained to the prospective respondents. Participants were informed that they could withdraw from the study at any time. Data were collected only from those who voluntarily agreed and provided written consent to participate in the study.

### 2.5. Data Analysis

Statistical analysis was conducted using SPSS, version 24.0 (IBM SPSS, New York, NY, USA). To check the normality of the variables, skewness, kurtosis, and the data’s histogram were all inspected. Multi-collinearity was examined by inspecting the coefficient of correlation, tolerance, variance inflation factor, and the linear relationships between variables. T-tests and analysis of variance were used to compare the level of health-promoting behaviors according to sample characteristics. The relationships among continuous variables were examined using Pearson’s correlation coefficients. Furthermore, a path analysis was conducted to test the hypothesized paths between the variables. Baron and Kenny’s recommendations [32] were applied in identifying the possible mediating effects of health self-efficacy between: (1) eHealth use and health-promoting behaviors, (2) eHealth literacy and health-promoting behaviors, and (3) informational support and health-promoting behaviors. The independent variables, mediators, and dependent variables in the path models are presented in Table 2. The procedures were conducted along the following steps: (1) confirmation was sought concerning whether the independent variable was significantly associated with the dependent variable through the use of simple regression analysis; (2) confirmation was sought concerning whether or not the independent variable was significantly associated with the mediator via the use of simple regression analysis; and (3) the dependent variable was regressed with both the independent variable and the mediator, thereby determining whether the mediator was significantly associated with the dependent variable, and if the β of the independent variable in Step 1 was reduced. The significance level was set at *p* < 0.05.

## 3. Results

### 3.1. Sample Characteristics

Table 3 describes the sample characteristics. The mean age was 75.56 years (SD = 5.98 years), with roughly 60% of respondents in their 70s. Approximately half (55.4%) of respondents were men. Approximately 18.8% of respondents reported as having no or elementary school level education, while 36% and 21.5% reported as having high school and college-level education, respectively. Among the 186 participants, 81.2% were not employed, and 68.8% reported moderate economic status. Some participants (15.1%) had no disease, while more than half (56%) had two or more complex chronic diseases. Among the respondents, 37.6% reported to having a good health status, while 17.2% reported having a poor health status. There were significant differences in the level of health-promoting behaviors according to sex (*p* = 0.025), perceived economic status (*p* = 0.026), and perceived health status (*p* < 0.001).

### 3.2. Relationships Between the Main Variables

Correlations among the main variables are presented in Table 4. People with higher eHealth use, better eHealth literacy, with greater informational support, and a higher level of health self-efficacy had better health-promoting behaviors. People with more eHealth use and informational support had higher health self-efficacy. Most variables showed a significant correlation in the predicted direction, except for cognition, eHealth literacy, and self-efficacy. A path model of eHealth literacy was excluded due to its non-significant relationships with other variables.

**Figure 1:**
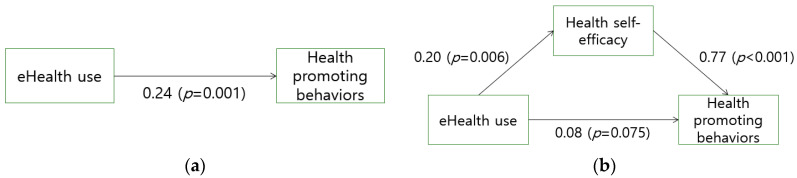
Relationship of eHealth use, health self-efficacy, and health-promoting behaviors. (**a**) Direct effect of eHealth on health-promoting behaviors; (**b**) Mediation of health self-efficacy between eHealth and health-promoting behaviors.

Figure 1 depicts how health self-efficacy mediates the relationship between eHealth use and health-promoting behaviors. eHealth use, an independent variable, was significantly associated with health-promoting behaviors and the dependent variable (β = 0.24, *p* = 0.001). The regression equation was:

(1) Y = 2.66(constant) + 0.24X + E1(error).

eHealth use was also significantly associated with health self-efficacy, a mediator (β = 0.20, *p* = 0.006). The regression Equation (2) was:

(2) M = 86.68(constant)+0.20X+E2(error).

Further analysis of the indirect effects of eHealth resource use was conducted based on the direct relationship between the main variables; when regressing health-promoting behaviors on eHealth use and health self-efficacy, the β value decreased from 0.24 to 0.08 and became non-significant (*p* = 0.075). The regression Equation (3) was:

(3) Y = 0.52(constant)+0.77M+E3(error).

This indicates that eHealth use only had an indirect effect on health-promoting behaviors mediated by self-efficacy.

**Figure 2:**
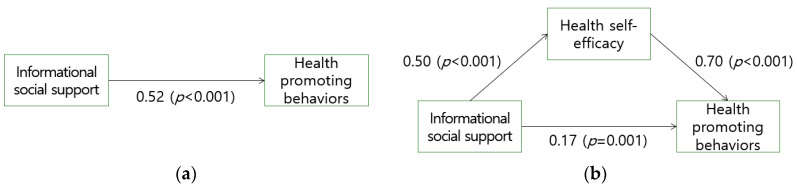
Relationship of informational social support, health self-efficacy, and health-promoting behaviors. (**a**) Direct effect of informational social support on health-promoting behaviors; (**b**) Mediation of health self-efficacy between informational social support and health-promoting behaviors.

Figure 2 depicts the mediating effect of health self-efficacy on informational support and health-promoting behaviors. Informational social support, an independent variable, was significantly associated with health-promoting behaviors and the dependent variable (β = 0.52, *p* < 0.001). The regression Equation (4) was

(4) Y=2.03(constant)+0.52X+E1(error).

Informational social support was also significantly associated with health self-efficacy, a mediator (β = 0.50, *p* < 0.006). The regression Equation (5) was

(5) M=67.14(constant)+0.50X+E2(error)

Further analysis of the indirect effects of informational social support was conducted based on the direct relationship between the main variables; when regressing health-promoting behaviors on informational social support and health self-efficacy, the β value decreased from 0.52 to 0.17. The regression Equation (6) was

(6) Y=0.53(constant)+0.17X+0.70M+E3(error)

This implies that health self-efficacy partially mediated the relationship between informational social support and health-promoting behaviors.

## 4. Discussion

Facilitating healthy aging is important in addressing issues related to the ever-growing older population. Health-promoting behaviors are especially important among older adults, as these individuals are more likely to have complex health conditions. This study identified the association between eHealth use, eHealth literacy, informational social support, and health-promoting behaviors, as well as the mediating effect had by health self-efficacy. This study found that informational social support had direct and indirect effects on health-promoting behaviors, while eHealth use, as mediated by self-efficacy, only had an indirect effect on health-promoting behaviors.

Self-efficacy displayed the greatest variance in the health-promoting behaviors of older adults. Accordingly, to facilitate healthy behavior among older individuals, it is important to provide appropriate interventions at improving self-efficacy among older adults. One of the factors which affects the health self-efficacy and health-promoting behaviors of older individuals is social support. In this study, it has been observed that informational social support had both direct and indirect effects, when mediated by health self-efficacy, on health-promoting behaviors. Informational social support—such as the provision of adequate information, advice, and instructions for health management—enhances self-efficacy which, in turn, facilitates health-promoting behaviors and healthy aging. Previous studies support these findings [33,34]; Wu and Sheng [34], for example, used a conceptual model [35] which demonstrated how social support networks impact health in order to verify the pathways by which social support—as mediated by self-efficacy—affected health-promoting behaviors. In light of these findings, healthcare professionals should assess informational support and provide effective interventions so as to improve support levels for older adults. Although social support may be implemented through educational programs and/or psychosocial interventions, a multicomponent social support program has been recommended in place of a single educational program [36]. As behavior beliefs such as self-efficacy and behavior plans are key precursors to health-related behaviors, a motivational and volitional multicomponent program—including education, psychotherapy, and self-management intervention—can help promote both precursors and actual positive health outcomes among older adults [37]. Thus, the role of healthcare professionals as educators, counselors, healthcare providers, and supporters is important in facilitating social support and health-promoting behaviors among older adults via comprehensive interventions.

Although eHealth was expected to have a direct effect, this study demonstrated that eHealth had an indirect effect on health-promoting behaviors, as mediated by health self-efficacy; however, no direct effect thereupon was observed. The effect of eHealth use on health-promoting behaviors became non-significant with borderline significance probability when health self-efficacy was introduced as a mediator. There is a possibility that our sample size was insufficient for detecting small effect sizes [38]. Despite the lack of a direct effect on health-promoting behaviors, eHealth can still be critical in improving health self-efficacy and management among older adults. Information, resources, and support are important aspects of engaging in health-promoting behaviors. Accessibility to these factors has been indicated as a key barrier facing older adults, particularly those with multimorbidity and the accompanying multitude of needs in managing their complex health conditions [39]. Older people reported using online health information to learn about their disease, medication, treatments, or healthy lifestyles [40]. In the wake of the development of various eHealth programs [10], online information and connections have become a convenient and financially viable approach available to older adults; thus, older adults need to be prepared for the increased use of the Internet in healthcare services and to adapt to the presence of eHealth in healthcare. Future studies should also develop strategies aimed at maintaining and improving access to health services for those who do not use eHealth resources.

eHealth and social support can synergistically promote healthy behaviors. A personalized behavioral intervention, as implemented via mHealth, was feasible and had effectively led older adults to promote and engage in physical activity [33]. The development of self-efficacy and improvements in health behaviors are not only enhanced via technology learning sessions—which help participants familiarize themselves with mobile devices, as well as self-monitoring and receiving feedback via a smartwatch—but also by utilizing appropriate social support resources through coaching and consultation conducted via phone. Since computer-mediated interactions are positively associated with perceived social support, communication facilitated by eHealth resources can also aid the health management of older adults [41]. Innovative health technology, when combined with social support from healthcare providers, may provide individualized interventions and encourage successful health-promoting behaviors in older adults. Healthcare providers can facilitate older adults’ health behaviors through interactions within the social support networks available to them. Thus, healthcare providers should assess older adults’ social networks—this includes caregivers who can help access eHealth resources, provide feedback, and search for credible information.

Considering the relationship between eHealth use and eHealth literacy, older adults with low levels of eHealth literacy may fear that they cannot screen and understand health-related materials and instructions and, consequently, may be reluctant to use eHealth resources otherwise available to them. As such, support is needed for older adults to effectively use information and resources available on the Internet so that they can both access and check the quality of online information and learn about their healthcare through educational interventions. However, in the present study, eHealth literacy was not associated with health self-efficacy, which is contrary to previous findings that reported a significant relationship between eHealth literacy and self-efficacy [42]. Individuals’ self-perceived capability to use health-related online sources did not seem to be related to confidence in adopting their use. A systematic review demonstrated an inconsistent relationship between health literacy and self-efficacy and explained the findings in terms of the measurement of health literacy itself [43]. Subsequently, it may be that the difference between objective and subjective measures of health literacy may affect the consistency of the results obtained. Older adults who report having high levels of health literacy may overestimate their capabilities to screen, understand, and analyze health information. Furthermore, among the three domains of health literacy, the domains of communicative (collaboration with healthcare providers) and critical health literacy (information analysis and decision making) may explain for the presence of greater variance in self-efficacy than that of functional health literacy (obtaining, processing, and understanding information), which eHEALS focuses on. These findings could not sufficiently explain the association of eHealth use, literacy, and self-efficacy; thus, further studies related to eHealth use among older adults are needed.

This study has several limitations. First, since the participants were recruited from senior welfare centers that provide older adults with various activities such as exercise programs, it is possible that older people who were more vulnerable to health promotion could not be included. Secondly, the study could not control for other factors, such as perceived benefits and barriers known to be associated with health-promoting behaviors. Thus, future studies should include these variables. Third, social support was evaluated using one of its sub-concepts: informational support. Finally, this study tested the two hypothetical path models; one model includes eHealth use, health self-efficacy and health-promoting behaviors, whereas the other model includes informational social support, health self-efficacy and health-promoting behaviors. This approach increases the likelihood of a type I error (a false positive). Further studies should use structural equation modeling to test a complete model for identifying the relationship between eHealth use, informational social support, health self-efficacy, and health-promoting behaviors.

## 5. Conclusions

Internet use among older individuals is increasing worldwide, and Internet-based health technologies are continuing to be developed alongside the aging of the population. This study identified the association of eHealth use and informational social support with health-promoting behaviors, as mediated by health self-efficacy. Healthcare providers should provide support to older adults to prepare them for the increased use of the Internet in healthcare services and to prepare them to be able to effectively use online information and resources available to them. Furthermore, it is also important for healthcare providers to facilitate the informational social support of older adults via comprehensive interventions. Encouraging eHealth use and informational social support are expected to facilitate the health-promoting behaviors of older adults by improving their health self-efficacy.

## Figures and Tables

**Table 1:** Instruments for each variable.

Variables	Instruments	Items	Scale
eHealth use	Internet Health Information-Seeking Behaviors [17]	13	5-point Likert scale
eHealth literacy	eHealth Literacy Scale [18]	8	5-point Likert scale
Informational social support	Social Support Scale [20]	25	5-point Likert scale
Health self-efficacy	Self-Rated Abilities for Health Practices [21]	24	5-point Likert scale
Health-promoting behaviors	Health-Promoting Lifestyle Profile Ⅱ [23]	52	4-point Likert scale

**Table 2:** Variables of regression equations in path models.

	Independent Variables (X)	Mediators (M)	Dependent Variables (Y)
Hypothetical path model 1	eHealth use	Health self-efficacy	Health-promoting behaviors
Hypothetical path model 2	eHealth literacy	Health self-efficacy	Health-promoting behaviors
Hypothetical path model 3	Informational social support	Health self-efficacy	Health-promoting behaviors

**Table 3:** Sample characteristics (N = 186).

Characteristics	Categories	n (%)	Mean (SD) of Health-Promoting Behaviors	t of F	*p*
Age	65~69	32 (17.2)	2.73 (0.54)	1.725	0.181
	70~79	111 (59.7)	2.89 (0.52)		
	80~	43 (23.1)	2.95 (0.48)		
Sex	Male	103 (55.4)	2.80 (0.53)	−2.260	0.025
	Female	83 (44.6)	2.97 (0.49)		
Educational level	No education or elementary school	35 (18.8)	3.0 (0.51)	1.673	0.174
	Middle school	44 (23.7)	2.86 (0.50)		
	High school	67 (36.0)	2.79 (0.57)		
	College~	40 (21.5)	2.96 (0.42)		
Occupational status	Yes	35 (18.8)	2.97 (0.54)	1.114	0.267
	No	151 (81.2)	2.86 (0.51)		
Perceived economic status	Good (a)	17 (9.1)	3.09 (0.48)	3.734	0.026 (a > c) ^†^
	So-so (b)	128 (68.8)	2.91 (0.49)		
	Bad (c)	41 (22.0)	2.71 (0.57)		
Marital status	Married	115 (61.8)	2.89 (0.48)	−0.361	0.718
	Others (single, divorced)	71 (38.2)	2.86 (0.58)		
Type of living	Living alone	52 (28.0) ^‡^	2.85 (0.60)	0.256	0.775
	Living with spouse	99 (53.2)	2.91 (0.50)		
	Living with children and/or spouse	35 (18.8)	2.86 (0.44)		
Number of comorbidities	0	28 (15.1)	2.94 (0.47)	0.473	0.756
	1	54 (29.0)	2.84 (0.53)		
	2	37 (19.9)	2.96 (0.43)		
	3	36 (19.4)	2.85 (0.59)		
	4~	31 (16.7)	2.83 (0.55)		
Perceived health status	Good (a)	70 (37.6)	3.11 (0.46)	12.57	<0.001 (a > b, c) ^†^
	So-so (b)	84 (45.2)	2.75 (0.46)		
	Bad (c)	32 (17.2)	2.71 (0.59)		

^†^ Scheffe test; ^‡^ Among 52, one respondent reported living with others who were not his/her family or relatives.

**Table 4:** Correlations between main variables (N = 186).

	Mean (SD)	X1	X2	X3	X4	X5
Cognitive function (X1)	25.95 (2.88)	1				
eHealth use (X2)	23.63 (13.24)	0.133	1			
eHealth literacy(X3)	25.35 (7.63)	0.211 **	0.584 **	1		
Informational social support (X4)	13.57 (4.32)	−0.015	0.085	0.146 *	1	
Health self-efficacy (X5)	92.47 (16.06)	−0.009	0.202 **	0.138	0.502 **	1
Health-promoting behaviors (X6)	2.88 (0.52)	−0.064	0.238 **	0.230 **	0.521 **	0.783 **

* *p* < 0.05; ** *p* < 0.01.
